# Alcohol and Medication Interactions

**Published:** 1999

**Authors:** Ron Weathermon, David W. Crabb

**Affiliations:** Ron Weathermon, Pharm.D., is an assistant professor at the School of Pharmacy and Pharmaceutical Sciences, Purdue University, Indianapolis, Indiana. David W. Crabb, M.D., is a professor in the Departments of Medicine and Biochemistry and Molecular Biology, Indiana University School of Medicine, Indianapolis, Indiana

**Keywords:** moderate AOD use, prescription drug, adverse drug interaction, drug metabolism, ethanol metabolism, cytochromes, liver, alcohol dehydrogenases, antibiotics, antidepressants, histamine H1 receptor blockaders, barbiturates, benzodiazepines, histamine H2 receptor blockaders, anti-inflammatory agents, opioids, warfarin, over-the-counter drug, literature review

## Abstract

Many medications can interact with alcohol, thereby altering the metabolism or effects of alcohol and/or the medication. Some of these interactions can occur even at moderate drinking levels and result in adverse health effects for the drinker. Two types of alcohol-medication interactions exist: (1) pharmacokinetic interactions, in which alcohol interferes with the metabolism of the medication, and (2) pharmacodynamic interactions, in which alcohol enhances the effects of the medication, particularly in the central nervous system (e.g., sedation). Pharmacokinetic interactions generally occur in the liver, where both alcohol and many medications are metabolized, frequently by the same enzymes. Numerous classes of prescription medications can interact with alcohol, including antibiotics, antidepressants, antihistamines, barbiturates, benzodiazepines, histamine H_2_ receptor antagonists, muscle relaxants, nonnarcotic pain medications and anti-inflammatory agents, opioids, and warfarin. In addition, many over-the-counter and herbal medications can cause negative effects when taken with alcohol.

Most people who consume alcohol, whether in moderate or large quantities, also take medications, at least occasionally. As a result, many people ingest alcohol while a medication is present in their body or vice versa. A large number of medications—both those available only by prescription and those available over the counter (OTC)—have the potential to interact with alcohol. Those interactions can alter the metabolism or activity of the medication and/or alcohol metabolism, resulting in potentially serious medical consequences. For example, the sedative effects of both alcohol and sedative medications can enhance each other (i.e., the effects are additive), thereby seriously impairing a person’s ability to drive or operate other types of machinery.

Most studies assessing alcohol-medication interactions focus on the effects of chronic heavy drinking. Relatively limited information is available, however, on medication interactions resulting from moderate alcohol consumption (i.e., one or two standard drinks^1^ per day). Researchers, physicians, and pharmacists must therefore infer potential medication interactions at moderate drinking levels based on observations made with heavy drinkers. In addition, moderate alcohol consumption may directly influence some of the disease states for which medications are taken (see sidebar, pp. 52–53, for further discussion of alcohol’s influences on various disease states). This article discusses alcohol absorption, distribution, and metabolism within the body; the sites where potential alcohol-medication interactions can occur; and possible adverse effects from various alcohol-medication combinations, including OTC or herbal products.

**Alcohol’s Influences on Various Disease States**

Many people who are being treated for chronic health problems, such as diabetes and high blood pressure (i.e., hypertension), consume alcohol, whether occasionally or regularly. As described in the main article, alcohol consumption, even at moderate levels, may interfere with the activities of many medications prescribed for such conditions. In addition, however, alcohol use may contribute to or exacerbate certain medical conditions.

***Diabetes***

In people with diabetes, control of the levels of the sugar glucose in the blood is severely impaired, either because these people lack the hormone insulin, which plays a central role in blood sugar regulation, or because their body does not respond appropriately to the insulin they produce. Alcohol consumption in diabetics can result either in higher-than-normal blood sugar levels (i.e., hyperglycemia) or in lower-than-normal blood sugar levels (i.e., hypoglycemia), depending on the patient’s nutritional status (Emanuele et al. 1998). Thus, long-term (i.e., chronic) alcohol consumption in well-nourished diabetics can lead to hyper-glycemia. Conversely, alcohol consumption in diabetics who have not eaten for a while and whose glucose resources are exhausted (i.e., who are in a fasting state) can induce hypoglycemia. Both hyperglycemia and hypoglycemia can have serious health consequences. Diabetes medications that substitute for or stimulate the body’s own insulin production (e.g., insulin or sulfonylureas) also may lead to hypoglycemia.

Alcohol-induced hypoglycemia occurs in the fasted state, when the diabetic’s blood sugar levels are already low and the body depends on the production of new glucose molecules (i.e., gluconeogenesis) to maintain sufficient blood glucose levels. Gluconeogenesis, which occurs in the liver, requires certain compounds whose levels are regulated by a substance called reduced nicotinamide adenine dinucleotide (NADH). Alcohol metabolism in the liver generates excessive NADH levels and thus reduces the levels of the compounds needed for gluconeogenesis, thereby contributing to a further drop in blood sugar levels. This response is particularly critical in diabetics taking medications that can cause hypoglycemia. Consequently, these patients should be advised to drink alcohol only with or shortly after meals.

Diabetics who consume alcohol also must be alert to the fact that the symptoms of mild intoxication closely resemble those of hypoglycemia. Accordingly, diabetics should check their blood glucose levels whenever they are uncertain about whether their symptoms are caused by hypoglycemia or alcohol intoxication (for additional recommendations for diabetics who consume alcohol, see the textbox). Finally, patients using certain diabetes medications (e.g., chlorpropamide) should be cautioned that the medications can cause a disulfiram-like reaction when alcohol is consumed.

**Preventing Alcohol-Induced Hypoglycemia**

Alcohol-consuming diabetic patients should consider the following general suggestions for preventing alcohol-induced hypoglycemia:

- Never consume alcohol without food or while in a fasting state.
- Consume only moderate amounts of alcohol (i.e., one or two bottles of beer, glasses of wine, or mixed drinks at one sitting), and drink no more than once or twice weekly.
- Allow 1.5 to 2 hours between drinks.
- Avoid sugar-containing drinks, and consume only light beer, dry wine, or drinks mixed with diet sodas.
- Check blood sugar levels if unsure whether certain body sensations (e.g., light-headedness) result from hypo-glycemia or alcohol effects.
- Be on the alert for alcohol hidden in prescription and over-the-counter medications.

***Hyperlipidemia***

In people with hyperlipidemia, the levels of fat molecules in the blood—particularly molecules called triglycerides—are higher than normal. This condition can be associated with an increased risk of various health problems, the most serious of which is cardiovascular disease. Alcohol consumption may exacerbate hyperlipidemia, because the same metabolic alcohol effects that inhibit gluconeogenesis also inhibit fat metabolism. As a result, the production of certain molecules called very low density lipoprotein (VLDL) particles is increased. Thus, people with elevated triglyceride levels in the blood should probably abstain from alcohol to determine if alcohol consumption is contributing to their elevated lipid levels.

***Hypertension***

Elevated blood pressure is a risk factor for cardiovascular disease, including heart attacks. Alcohol is known to cause a dose-dependent elevation in blood pressure (Beilin 1995). Researchers do not yet know exactly what levels of alcohol consumption cause hypertension (for more information, see the article by Klatsky, pp. 15–23). However, all patients who are diagnosed with high blood pressure should be questioned regarding their alcohol intake before being started on antihypertensive therapy. In some of those patients, cessation of drinking alone may reduce blood pressure and thus obviate the need for pharmacological treatment. Furthermore, patients taking certain kinds of cardiac medications (e.g., isosorbide [Isordil^®^ and Ismo^®^], terazosin [Hytrin^®^], doxazosin [Cardura^®^]) should be warned that alcohol consumption in combination with those medications may cause lower-than-normal blood pressure. These important potential risks associated with even moderate alcohol consumption (i.e., one or two standard drinks^1^ per day) must be considered when discussing the cardiovascular benefits associated with moderate drinking (e.g., reduced risk of heart attacks and certain kinds of strokes.)

***Hepatitis C Infection***

Infection with the hepatitis C virus, which can result in serious and even fatal liver damage, is common in the United States and around the world. The only effective treatment to date involves a substance called interferon-α, often in combination with an agent called ribavirin, and has a cure rate of approximately 40 percent. Heavy alcohol use in patients infected with hepatitis C accelerates the rate of liver damage and increases the risk of cirrhosis. Moreover, heavy alcohol use appears to reduce the number of hepatitis C-infected people who respond to treatment with interferon-α. Researchers do not yet know how alcohol consumption exacerbates disease progression and interferes with treatment. Nevertheless, people infected with the hepatitis C virus probably should avoid using alcohol, particularly during interferon-α treatment.

— Ron Weathermon and David W. Crabb

1A standard drink is defined as one 12-ounce can of beer or bottle of wine cooler, one 5-ounce glass of wine, or 1.5 ounces of distilled spirits and is equivalent to approximately 0.5 ounce, or 12 grams (g), of pure alcohol.

ReferencesBeilinLJAlcohol and hypertensionClinical and Experimental Pharmacology and Physiology221851881995755441110.1111/j.1440-1681.1995.tb01977.xEmanueleNVSwadeTFEmanueleMAConsequences of alcohol use in diabeticsAlcohol Health & Research World223211219199815706798PMC6761899

## Alcohol Absorption, Distribution, and Metabolism

### Gastrointestinal Absorption and Metabolism

When alcohol is ingested through the mouth, a small amount is immediately broken down (i.e., metabolized) in the stomach. Most of the remaining alcohol is then absorbed into the bloodstream from the gastrointestinal tract, primarily the stomach and the upper small intestine. Alcohol absorption occurs slowly from the stomach but rapidly from the upper small intestine. Once absorbed, the alcohol is transported to the liver through the portal vein. A portion of the ingested alcohol is metabolized during its initial passage through the liver; the remainder of the ingested alcohol leaves the liver, enters the general (i.e., systemic) circulation, and is distributed throughout the body’s tissues.

**Figure 1:**
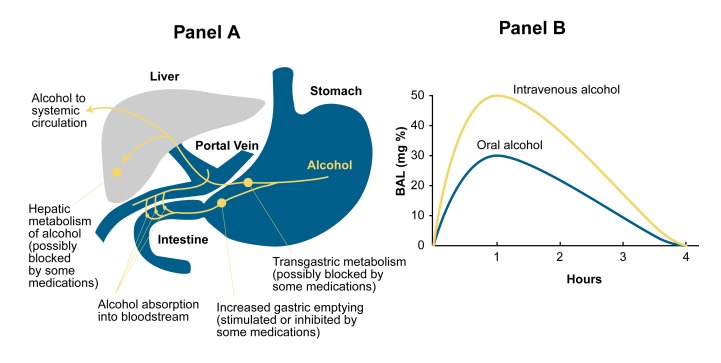
Schematic representation of first-pass metabolism. (A) Alcohol ingested through the mouth reaches the stomach, where a portion is metabolized by the enzyme alcohol dehydrogenase (ADH). The remaining alcohol enters the intestine, where most of the remainder is absorbed into the bloodstream and enters the portal vein that leads to the liver. In the liver, part of the alcohol is metabolized by ADH or cytochrome P450. The remaining alcohol enters the general (i.e., systemic) circulation and eventually is transported back to the liver and metabolized there. The metabolism of alcohol in the stomach or during the first passage through the liver after absorption from the intestine is called first-pass metabolism. (B) Changes in blood alcohol levels (BALs) after oral alcohol ingestion and after intravenous administration of the same alcohol dose. The difference in BALs achieved with both administration routes (i.e., the amount by which the BAL is lower after oral ingestion) represents that portion of the ingested alcohol that has been broken down by first-pass metabolism before reaching the systemic circulation.

Alcohol metabolism (or the metabolism of any other substance) that occurs in the gastrointestinal tract and during the substance’s initial passage through the liver is called “ first-pass metabolism” (see figure 1). For example, the mucosa lining the stomach contains enzymes that can metabolize alcohol as well as other substances; some of those enzymes, including alcohol dehydrogenase (ADH) and cytochrome P450 are described in more detail in the section “Alcohol Metabolism in the Liver.”

The contribution of stomach (i.e., gastric) enzymes to first-pass alcohol metabolism, however, is controversial. Whereas some researchers have proposed that gastric enzymes play a major role in first-pass metabolism (Lim et al. 1993), other investigators consider the liver to be the primary site of first-pass metabolism (Levitt and Levitt 1998). Furthermore, some gender differences appear to exist in the overall extent of, and in the contribution of, gastric enzymes to first-pass metabolism. For example, the extent of first-pass metabolism is less in women than in men and some studies also have found lower gastric ADH activity in women (Thomasson 1995).

First-pass metabolism is readily detectable after consumption of low alcohol doses^2^ that leave the stomach slowly (e.g., because they have been consumed with a meal). Thus, under such conditions of delayed gastric emptying, more alcohol can be metabolized in the stomach or absorbed slowly from the stomach and transported to the liver for first-pass metabolism.

In general, probably only a small fraction (perhaps 10 percent) of ingested alcohol is eliminated from the body by first-pass metabolism after consumption of low doses of alcohol. As alcohol ingestion increases, the amount of alcohol eliminated by first-pass metabolism becomes an even smaller fraction of the total amount of alcohol consumed. Some researchers have suggested, however, that some medications can block first-pass metabolism, resulting in blood alcohol levels (BALs) that are higher than normal for a given alcohol dose. For example, people taking medications that can inhibit ADH activity—such as aspirin and certain medications used to treat ulcers and heartburn (i.e., H_2_ receptor antagonists, such as cimetidine [Tagamet^®^ ], nizatidine [Axid^®^ ] and ranitidine [Zantac^®^ ])—experience reduced first-pass metabolism (Caballaria et al. 1991; Roine et al. 1990). Similarly, medications that accelerate gastric emptying (e.g., the stomach medications metoclopramide [Reglan^®^ ] and cisapride [Propulsid^®^ ] and the antibiotic erythromycin) may reduce first-pass metabolism in the stomach.

The contribution of bacteria living in the large intestine (i.e., colon) to gastrointestinal alcohol metabolism is still controversial. Laboratory experiments have demonstrated that these bacteria can metabolize alcohol*.* In addition, a breakdown product of alcohol (i.e., acetaldehyde) is generated in the colon after alcohol administration. Finally, studies in rats found that animals treated with an antibiotic to reduce the number of bacteria in the colon showed a reduced alcohol elimination rate compared with untreated rats (Nosova et al. 1999). If these research findings also apply to humans, alcohol elimination may be delayed in people taking certain antibiotics that are active against colonic bacteria.

### Alcohol’s Distribution in the Body

Alcohol that has not been eliminated by first-pass metabolism enters the systemic circulation and is distributed throughout the body water (i.e., the blood and the watery fluid surrounding and inside the cells). Alcohol does not dissolve in fat tissues. The proportion of body water and body fat differs between men and women and between young and old people; women and older people generally have more body fat and less body water than do men and younger people. As a result, alcohol distribution throughout the body depends on a person’s gender and age.

Differences in alcohol distribution patterns also affect the BALs achieved with a given alcohol dose (Thomasson 1995). Thus, women, whose lower body water creates a smaller fluid volume in which the alcohol is distributed, tend to achieve higher BALs than do men after consuming the same amount of alcohol. The normal loss of lean body weight and increase in body fat that occurs with aging has a similar effect on BALs. The potentially higher BALs can exaggerate alcohol-medication interactions in both women and older people. Aside from this effect of gender and age on BALs, researchers have not reported any other major gender- or age-related differences in susceptibility to alcohol-medication interactions. However, this issue still requires further investigation.

### Alcohol Metabolism in the Liver

**Figure 2:**
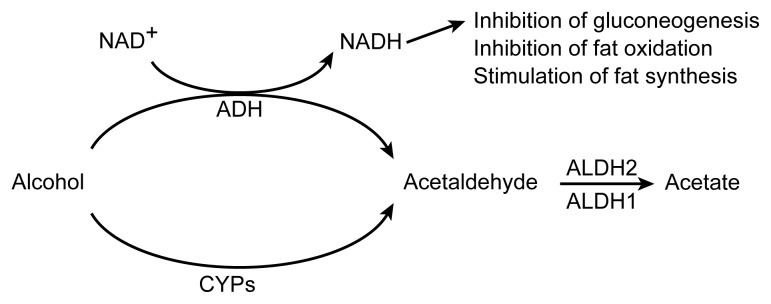
Alcohol metabolism in the liver. Alcohol is broken down to acetaldehyde either by alcohol dehydrogenase (ADH) or cytochrome P450 (CYP). The acetaldehyde then is broken down to acetic acid and water by two variants of the enzyme aldehyde dehydrogenase (ALDH). Alcohol metabolism by ADH generates a byproduct called reduced nicotinamide adenine dinucleotide (NADH). Excessive NADH levels can inhibit glucose production (i.e., gluconeogenesis) and breakdown (i.e., oxidation) of fat molecules as well as stimulate production of fat molecules.

The liver is the primary site of alcohol metabolism. Alcohol circulating in the blood is transported to the liver, where it is broken down by several enzymes, the most important of which are ADH and cytochrome P450 (figure 2). The activities of these enzymes may vary from person to person, contributing to the observed variations in alcohol elimination rates among individuals (Martin et al. 1985).

ADH converts alcohol into acetaldehyde in a reaction called oxidation. Acetaldehyde, which is a toxic substance that may contribute to many of alcohol’s adverse effects, is broken down further by an enzyme called aldehyde dehydrogenase (ALDH). (The function of ALDH is discussed in more detail in the following section.) Several ADH variants (i.e., isozymes) exist, which differ in their activity when studied in the laboratory. In humans, however, the effect of different ADH isozymes on alcohol elimination is small (Thomasson 1995). Although different ADH variants are associated with different risks of developing alcoholism, no studies to date have researched the effects of these isozymes on a person’s susceptibility to alcohol-medication interactions.

In contrast to ADH, the alcohol-metabolizing enzyme cytochrome P450—also called microsomal ethanol oxidizing system (MEOS) (Lieber 1994)—plays a central role in alcohol-medication interactions. Cytochrome P450 actually is a system consisting of two enzymes, one called cytochrome P450 reductase and another one called CYP2E1, which are both embedded in the membrane of a cell component called the endoplasmic reticulum.^3^ In addition to alcohol, CYP2E1 can metabolize numerous compounds, including acetaldehyde, the pain medication acetaminophen, the antibiotic isoniazid, and the barbiturate phenobarbital. Accordingly, CYP2E1 plays an important role in many alcohol-medication interactions.

**Figure 3:**
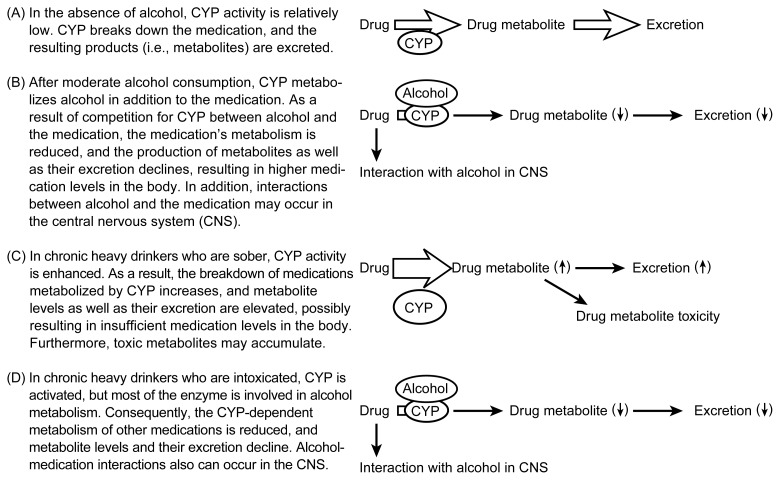
Potential alcohol-medication interactions involving cytochrome P450 enzymes (CYP) in the liver.

In people consuming alcohol only occasionally, CYP2E1 metabolizes only a small fraction of the ingested alcohol. Chronic heavy drinking, however, can increase CYP2E1 activity up to tenfold, resulting in a substantial increase in the proportion of alcohol that is metabolized by this enzyme rather than by ADH (figure 3) (Lieber 1994). The effect of lower levels of alcohol consumption on CYP2E1 activity is unknown. Because CYP2E1 also metabolizes several medications, alcoholics, in whom CYP2E1 activity is enhanced, exhibit increased metabolic rates for those medications when they are sober. When those alcoholics are intoxicated, however, the alcohol in their system competes with the medication for metabolism by CYP2E1. As a result, the breakdown of the medication is slowed. With many medications, increased or decreased metabolic rates can have adverse or even fatal consequences. With increased metabolic rates, the medication’s concentration in the body may be too low or may decline too fast for it to be effective. Conversely, decreased metabolic rates may result in the accumulation of higher drug concentrations over longer periods of times, which may result in harmful overdoses.

Wide variation exists among people in both CYP2E1 activity and metabolic rates for medications broken down by this enzyme (e.g., acetaminophen and chlorzoxasone, a medication used to relieve muscle pain). Some of this variation may be genetically determined, although the specific underlying mechanism is unknown (Carriere et al. 1996). A person’s CYP2E1 activity level, however, could influence his or her susceptibility to alcohol-medication interactions involving this enzyme. For example, in a person with innately low metabolic rates, a further decrease in metabolism when alcohol is consumed would affect medication levels (and thus the potential for adverse effects or interactions with alcohol) to a greater extent than in a person with innately high metabolic rates.

In addition to CYP2E1, at least two other cytochrome enzymes that metabolize various medications (i.e., CYP3A4 and CYP1A2) also can break down alcohol (Salmela et al. 1998). Moreover, the amounts of various enyzmes of the cytochrome CYP3A family (including CYP3A4) can increase from alcohol consumption (Niemela et al. 1998). Thus, potential interactions also exist between alcohol and medications metabolized by these cytochromes.

### Acetaldehyde Metabolism in the Liver

As mentioned in the previous section, alcohol breakdown by ADH generates acetaldehyde, which, in turn, is metabolized further by ALDH. Two major types of ALDH (i.e., ALDH1 and ALDH2) exist, which are located in different regions of the cell. ALDH1 requires relatively high acetaldehyde concentrations in the cell to be active, whereas ALDH2 is active at extremely low acetaldehyde levels. Accordingly, ALDH2 may play a particularly important role in acetaldehyde breakdown after moderate alcohol consumption.

The significance of ALDH2 activity in alcohol and acetaldehyde metabolism is further supported by an inborn variation in alcohol metabolism that occurs primarily in people of Asian heritage but which is rare among Caucasians. After consuming alcohol, many Asian people experience an unpleasant “ flushing” reaction that can include facial flushing, nausea, and vomiting. These symptoms are caused by acetaldehyde accumulation in the body. Thus, following alcohol consumption, acetaldehyde levels in people susceptible to the flushing reaction may be 10 to 20 times higher than in people who do not experience flushing. Researchers have noted that approximately 40 percent of Asians lack ALDH2 activity because they have inherited one or two copies of an inactive variant of the gene that produces ALDH2 (Goedde et al. 1989). Most of these individuals flush when they consume alcohol. These observations imply that ALDH2 plays a crucial role in maintaining low acetaldehyde levels during alcohol metabolism. Consequently, even inadvertent alcohol administration to people of Asian heritage (who may have inherited an inactive ALDH2 gene) can cause unpleasant reactions. Thus, the potential flushing response should be an important concern for physicians and patients, because many prescription and OTC medications contain substantial amounts of alcohol (see table 1). Physicians and pharmacists therefore must be alert to the possibility that Asian patients may be intolerant of these medications.

Several medications can inhibit even active ALDH molecules (both ALDH1 and ALDH2), thereby inducing a flushing reaction in all people who consume alcohol after taking those medications. In fact, this medication effect is exploited in alcoholism treatment. The medication disulfiram (Antabuse^®^ ), which inhibits mainly ALDH1 but also ALDH2, is given to alcoholics trying to quit drinking. If the alcoholic drinks alcohol after taking disulfiram, he or she will experience a severe flushing reaction. The experience of such an unpleasant reaction, or even the expectation that this reaction will occur if alcohol is consumed, can help many alcoholics achieve and maintain abstinence. Moderate drinkers are not likely to be treated with disulfiram; however, many other medications (and certain toxic substances) also can induce disulfiram-like reactions when combined with alcohol (see table 2). For example, the commonly prescribed diabetes medication chlorpropamide and the antibiotics cefotetan and metronidazole can induce disulfiram-like reactions, even after ingestion of only small alcohol amounts. These reactions not only are unpleasant but also can result in serious medical consequences. For example, flushing is associated with a widening (i.e., dilation) of the blood vessels, low blood pressure, and rapid heartbeat, all of which can be dangerous in patients with coronary artery disease. Patients taking medications that can induce disulfiram-like reactions therefore should be advised not to drink alcohol.

### Alcohol’s Effects on Liver Metabolism

In addition to influencing the metabolism of many medications by activating cytochrome P450 enzymes in the liver, alcohol and its metabolism cause other changes in the liver’s ability to eliminate various substances from the body. Thus, alcohol metabolism affects the liver’s redox state and glutathione levels. The term “redox state” refers to the concentrations of two substances in the cells—nicotinamide adenine dinucleotide (NAD^+^) and reduced NAD^+^ (NADH)—that are needed for the functioning of many enzymes. Alcohol metabolism by ADH results in the conversion of NAD^+^ into NADH, thereby increasing the liver’s NADH levels (see figure 2). Elevated NADH levels, in turn, stimulate the generation of fat molecules and interfere with the ability of other liver enzymes to break down fat molecules and produce the sugar glucose. Through these metabolic changes, alcohol metabolism can substantially affect the body’s general metabolism and functioning. Furthermore, elevated NADH levels may prevent the liver from generating UDP-glucuronic acid, a substance that must be attached to various medications before they can be excreted from the body.

Glutathione is an antioxidant, an agent that prevents certain highly reactive, oxygen-containing molecules (i.e., reactive oxygen species) from damaging the cells. Both alcohol metabolism and the metabolism of certain medications can generate reactive oxygen species, thereby inducing a state called oxidative stress in the cells. At the same time, heavy alcohol consumption reduces the amount of glutathione in liver cells, particularly in the mitochondria (i.e., the cell components where most of the cell’s energy is generated). Consequently, the cell’s protective mechanisms against oxidative stress are impaired, and cell death may result. Furthermore, reduced glutathione levels increase the liver’s susceptibility to damage caused by toxic breakdown products of some medications (e.g., acetaminophen and isoniazid).

## Common Alcohol-Medication Interactions

### Mechanisms of Alcohol-Medication Interactions

Interactions between alcohol and a medication can occur in a variety of situations that differ based on the timing of alcohol and medication consumption. For example, such interactions can occur in people who consume alcohol with a meal shortly before or after taking a medication or who take pain medications after drinking to prevent a hangover. Alcohol-medication interactions fall into two general categories: pharmacokinetic and pharmacodynamic. Pharmacokinetic interactions are those in which the presence of alcohol directly interferes with the normal metabolism of the medication. This interference can take two forms, as follows:

- The breakdown and excretion of the affected medications are delayed, because the medications must compete with alcohol for breakdown by cytochrome P450. This type of interaction has been described mostly for metabolic reactions involving CYP2E1, but it also may involve CYP3A4 and CYP1A2 (Salmela et al. 1998).
- The metabolism of the affected medications is accelerated, because alcohol enhances the activity of medication-metabolizing cytochromes. When alcohol is not present simultaneously to compete for the cytochromes, increased cytochrome activity results in an increased elimination rate for medications that these enzymes metabolize.

Pharmacodynamic alcohol-medication interactions do not involve enzyme inhibition or activation, but rather refer to the additive effects of alcohol and certain medications. In this type of interaction, which occurs most commonly in the central nervous system (CNS), alcohol alters the effects of the medication without changing the medication’s concentration in the blood. With some medications (e.g., barbiturates and sedative medications called benzodiazepines), alcohol acts on the same molecules inside or on the surface of the cell as does the medication. These interactions may be synergistic—that is, the effects of the combined medications exceed the sum of the effects of the individual medications. With other medications (e.g., antihistamines and antidepressants) alcohol enhances the sedative effects of those medications but acts through different mechanisms from those agents.

### Specific Alcohol-Medication Interactions

This section describes different classes of medications and their interactions with alcohol (see table 3). The potential for the occurrence and relevance of alcohol-medication interactions in moderate drinkers may differ, however, between pharmacokinetic and pharmacodynamic interactions. The number of potential pharmacokinetic interactions with alcohol is great, because the various cytochrome P450 enzymes metabolize many medications.^4^ However, many of the pharmacokinetic interactions discussed here were first discovered in heavy drinkers or alcoholics or were studied in animals given large alcohol doses in their diet. Although the potential for such effects certainly exists even after low alcohol consumption, researchers have not yet demonstrated the occurrence and relevance of those effects in moderate drinkers. Conversely, pharmacodynamic interactions can occur with intermittent alcohol consumption and even after a single episode of drinking. Accordingly, those interactions clearly pertain to moderate drinkers.

#### Antibiotics

The package inserts for most antibiotics include a warning for patients to avoid using alcohol with those medications. The rationale for these warnings is not entirely clear, however, because only a few antibiotics appear to interact with alcohol. For example, although some antibiotics induce flushing, most antibiotics do not. The antibiotic erythromycin may increase alcohol absorption in the intestine (and, consequently, increase BALs) by accelerating gastric emptying. Furthermore, people taking the antituberculosis drug isoniazid should abstain from alcohol, because isoniazid can cause liver damage, which may be exacerbated by daily alcohol consumption. Aside from these effects, however, moderate alcohol consumption probably does not interfere with antibiotic effectiveness. Possibly, concerns regarding the concurrent use of alcohol and antibiotics grew from research findings indicating that heavy alcohol use can impair the function of certain immune cells and that alcoholics are predisposed to certain infections. These effects, however, are unlikely to occur in moderate drinkers.

#### Antidepressants

Several classes of antidepressant medications exist, including tricyclic antidepressants (TCAs), selective serotonin reuptake inhibitors (SSRIs), monoamine oxidase (MAO) inhibitors, and atypical antidepressants. These classes differ in their mechanism of action in that they affect different brain chemicals. All types of antidepressants, however, have some sedative as well as some stimulating activity.

TCAs with a higher ratio of sedative-to-stimulant activity (i.e., amitriptyline, doxepin, maprotiline, and trimipramine) will cause the most sedation. Alcohol increases the TCAs’ sedative effects through pharmacodynamic interactions. In addition, alcohol consumption can cause pharmacokinetic interactions with TCAs. For example, alcohol appears to interfere with the first-pass metabolism of amitriptyline in the liver, resulting in increased amitriptyline levels in the blood*.* In addition, alcohol-induced liver disease further impairs amitriptyline breakdown and causes significantly increased levels of active medication in the body (i.e., increased bioavailability). High TCA levels, in turn, can lead to convulsions and disturbances in heart rhythm.

SSRIs (i.e., fluvoxamine, fluoxetine, paroxetine, and sertraline), which are currently the most widely used anti-depressants, are much less sedating than are TCAs. In addition, no serious interactions appear to occur when these agents are consumed with moderate alcohol doses (Matilla 1990). In fact, SSRIs have the best safety profile of all antidepressants, even when combined in large quantities with alcohol (e.g., in suicide and overdose situations).

Conversely, people taking MAO inhibitors or atypical antidepressants can experience adverse consequences when simultaneously consuming alcohol. Thus, MAO inhibitors (e.g., phenelzine and tranylcypromine) can induce severe high blood pressure if they are consumed together with a substance called tyramine, which is present in red wine. Accordingly, people taking MAO inhibitors should be warned against drinking red wine. The atypical antidepressants (i.e., nefazodone and trazodone) may cause enhanced sedation when used with alcohol.

#### Antihistamines

These medications, which are available both by prescription and OTC, are used in the management of allergies and colds. Antihistamines may cause drowsiness, sedation, and low blood pressure (i.e., hypotension), especially in elderly patients (Dufour et al. 1992). Through pharmacodynamic interactions, alcohol can substantially enhance the sedating effects of these agents and may thereby increase, for example, a person’s risk of falling or impair his or her ability to drive or operate other types of machinery. As a result of these potential interactions, warning labels on OTC antihistamines caution patients about the possibility of increased drowsiness when consuming the medication with alcohol. Newer antihistamines (i.e., certrizine and loratidine) have been developed to minimize drowsiness and sedation while still providing effective allergy relief. However, these newer medications may still be associated with an increased risk of hypotension and falls among the elderly, particularly when combined with alcohol. Consequently, patients taking nonsedating antihistamines still should be warned against using alcohol.

#### Barbiturates

These medications are sedative or sleep-inducing (i.e., hypnotic) agents that are frequently used for anesthesia. Phenobarbital, which is probably the most commonly prescribed barbiturate in modern practice, also is used in the treatment of seizure disorders. Phenobarbital activates some of the same molecules in the CNS as does alcohol, resulting in pharmacodynamic interactions between the two substances. Consequently, alcohol consumption while taking phenobarbital synergistically enhances the medication’s sedative side effects. Patients taking barbiturates therefore should be warned not to perform tasks that require alertness, such as driving or operating heavy machinery, particularly after simultaneous alcohol consumption.

In addition to the pharmacodynamic interactions, pharmacokinetic interactions between alcohol and phenobarbital exist, because alcohol inhibits the medication’s breakdown in the liver. This inhibition results in a slower metabolism and, possibly, higher blood levels of phenobarbital. Conversely, barbiturates increase total cytochrome P450 activity in the liver and accelerate alcohol elimination from the blood (Bode et al. 1979). This acceleration of alcohol elimination probably does not have any adverse effect.

#### Benzodiazepines

Like barbiturates, benzodiazepines (BZDs) are classified as sedative-hypnotic agents and act through the same brain molecules as do barbiturates. Accordingly, as with barbiturates, concurrent consumption of BZDs and moderate amounts of alcohol can cause synergistic sedative effects, leading to substantial CNS impairment. It is worth noting that both barbiturates and benzodiazepines can impair memory, as can alcohol. Consequently, the combination of these medications with alcohol would exacerbate this memory-impairing effect. In fact, this effect sometimes is exploited by mixing alcoholic beverages with BZDs, such as the rapid-acting flunitrazepam (Rohypnol^®^ ), an agent implicated in date rape (Simmons and Cupp 1998). In addition, the metabolism of certain BZDs involves cytochrome P450, leading to the alcohol-induced changes in metabolism described earlier in this article.

#### Histamine H_2_ Receptor Antagonists (H_2_RAs)

As mentioned earlier in this article, H_2_RAs (e.g., cimetidine, ranitidine, nizatidine, and famotidine), which reduce gastric acid secretion, are used in the treatment of ulcers and heartburn. These agents reduce ADH activity in the stomach mucosa (Caballeria et al. 1991), and cimetidine also may increase the rate of gastric emptying. As a result, alcohol consumed with cimetidine undergoes less first-pass metabolism, resulting in increased BALs. For example, in a study of people who consumed three or four standard drinks over 135 minutes while taking cimetidine, BALs rose higher and remained elevated for a longer period of time than in people not taking cimetidine (Lieber 1997; Gupta et al. 1995). Not all H_2_RAs, however, exert the same effect on BALs when taken with alcohol. Thus, cimetidine and ranitidine have the most pronounced effect, nizatidine has an intermediate effect, and famotidine appears to have no effect (i.e., appears not to interact with alcohol).^5^ In addition, because women generally appear to have lower first-pass metabolism of alcohol, they may be at less risk for adverse interactions with H_2_RAs.

#### Muscle Relaxants

Several muscle relaxants (e.g., carisoprodol, cyclobenzaprine, and baclofen), when taken with alcohol, may produce a certain narcotic-like reaction that includes extreme weakness, dizziness, agitation, euphoria, and confusion. For example, carisoprodol is a commonly abused and readily available prescription medication that is sold as a street drug. Its metabolism in the liver generates an anxiety-reducing agent that was previously marketed as a controlled substance (meprobamate). The mixture of carisoprodol with beer is popular among street abusers for creating a quick state of euphoria.

### Nonnarcotic Pain Medications and Anti-Inflammatory Agents

Many people frequently use nonnarcotic pain medications and anti-inflammatory agents (e.g., aspirin, acetaminophen, or ibuprofen) for headaches and other minor aches and pains. In addition, arthritis and other disorders of the muscles and bones are among the most common problems for which older people consult physicians (Adams 1995). Nonsteroidal anti-inflammatory drugs (NSAIDs) (e.g., ibuprofen, naproxen, indomethacin, and diclofenac) and aspirin are commonly prescribed or recommended for the treatment of these disorders and are purchased OTC in huge amounts. Several potential interactions exist between alcohol and these agents, as follows:

- NSAIDs have been implicated in an increased risk of ulcers and gastrointestinal bleeding in elderly people. Alcohol may exacerbate that risk by enhancing the ability of these medications to damage the stomach mucosa (Adams 1995).^6^
- Aspirin, indomethacin, and ibuprofen cause prolonged bleeding by inhibiting the function of certain blood cells involved in blood clot formation. This effect also appears to be enhanced by concurrent alcohol use (Deykin et al. 1982).
- Aspirin has been shown to increase BALs after small alcohol doses, possibly by inhibiting first-pass metabolism (Roine et al. 1990).

An important pharmacokinetic interaction between alcohol and acetaminophen can increase the risk of acetaminophen-related toxic effects on the liver. Acetaminophen breakdown by CYP2E1 (and possibly CYP3A) results in the formation of a toxic product that can cause potentially life-threatening liver damage. As mentioned earlier, heavy alcohol use enhances CYP2E1 activity. In turn, enhanced CYP2E1 activity increases the formation of the toxic acetaminophen product. To prevent liver damage, patients generally should not exceed the maximum doses recommended by the manufacturers (i.e., 4 grams, or up to eight extra-strength tablets of acetaminophen per day). In people who drink heavily or who are fasting (which also increases CYP2E1 activity), however, liver injury may occur at doses as low as 2 to 4 grams per day. The specific drinking levels at which acetaminophen toxicity is enhanced are still unknown. Because acetaminophen is easily available OTC, however, labels on the packages warn people about the potentially dangerous alcohol-acetaminophen combination. Furthermore, people should be aware that combination cough, cold, and flu medications may contain aspirin, acetaminophen, or ibuprofen, all of which might contribute to serious health consequences when combined with alcohol.

#### Opioids

Opioids are agents with opium-like effects (e.g., sedation, pain relief, and euphoria) that are used as pain medications. Alcohol accentuates the opioids’ sedating effects. Accordingly, all patients receiving narcotic prescriptions should be warned about the drowsiness caused by these agents and the additive effects of alcohol. Overdoses of alcohol and opioids are potentially lethal because they can reduce the cough reflex and breathing functions; as a result, the patients are at risk of getting foods, fluids, or other objects stuck in their airways or of being unable to breathe.

Certain opioid pain medications (e.g., codeine, propoxyphene, and oxycodone) are manufactured as combination products containing acetaminophen. These combinations can be particularly harmful when combined with alcohol because they provide “ hidden” doses of acetaminophen. As described in the previous section, alcohol consumption may result in the accumulation of toxic breakdown products of acetaminophen. Therefore, patients using opioid-acetaminophen combination products should be cautioned about restricting the total amount of acetaminophen they ingest daily (i.e., they should not take regular acetaminophen in addition to the combination product).

#### Warfarin

The anticoagulant warfarin is used for the prevention of blood clots in patients with irregular heart rhythms or artificial heart valves; it is also used to treat clots that form in extremities such as legs, arms, or sometimes the lungs. Its anticoagulant effect is acutely altered by even small amounts of alcohol. In people taking warfarin and ingesting a few drinks in one sitting, anticlotting effects may be stronger than necessary for medical purposes, placing these people at risk for increased bleeding. This excessive warfarin activity results from alcohol-related inhibition of warfarin metabolism by cytochrome P450 in the liver (Lieber 1994). Conversely, in people who chronically drink alcohol, long-term alcohol consumption activates cytochrome P450 and, consequently, warfarin metabolism. As a result, warfarin is broken down faster than normal, and higher warfarin doses are required to achieve the desired anticoagulant effect. Thus, alcohol consumption can result in dangerously high or insufficient warfarin activity, depending on the patient’s drinking pattern. Therefore, patients taking warfarin generally should avoid alcohol.

## Moderate Alcohol Consumption and OTC or Herbal Medications

Use of OTC medications is widespread among the general population. According to a recent survey, 85 percent of adults ages 18 and older have used OTC pain relievers at least once, and up to 34 percent use OTC pain relievers on a weekly basis, often without consulting a pharmacist. Furthermore, a recent scientific panel convened by the American Pharmaceutical Association (1997) reported that although adults frequently use OTC medications, many consumers fail to read the product warning labels. Finally, consumers frequently are unaware of the type of medication they take (e.g., NSAID or analgesic). For example, only one in three adults are familiar with the product names acetaminophen, aspirin, or ibuprofen and are able to link these product names to specific brand names. As a result, many consumers are not fully aware of the potential risks of taking these products, particularly in combination with other prescription medications or alcohol.

Another factor contributing to an increasing risk of medication-medication or alcohol-medication interactions is that many medications that previously were available only by prescription (e.g., H_2_RAs and NSAIDs) are gaining OTC status. OTC marketing strategies, however, often lead the consumer to think that these medications are safe to use on an “as-needed” basis, even though they can be potentially dangerous when used with alcohol. For example, the message that “acid blocker” medications can be used before or during a spicy meal to prevent heartburn symptoms may lead consumers to believe that this practice is also acceptable when they drink alcohol with their meal.

Not only the combination of alcohol and OTC products but also the amount of alcohol contained in various OTC products can be dangerous (see table 1). Alcohol concentrations in these products can be substantial; mouthwashes and cough syrups tend to have the highest alcohol contents. In an effort to control the alcohol amounts in these products and promote safety, the Food and Drug Administration’s (FDA’s) OTC-drugs advisory committee has adopted the following limits on the amount of alcohol considered appropriate:

- For children under age 6, products should be “alcohol free” (i.e., have an alcohol content of 0.5 percent or less).
- For children ages 6 to 12, the alcohol content should range between 0.5 and 5 percent.
- For people over age 12, the alcohol content should not exceed 5 to 10 percent.

These levels represent only guidelines, however, and are not enforced by the FDA. The manufacturers of OTC products have agreed to maintain certain standards to keep their products as close to these suggestions as possible. Nevertheless, higher alcohol concentrations are considered acceptable in certain products, such as herbal medications, because alcohol often is needed to extract and dissolve organic substances from plants.

Herbal medications currently are widely used, and many people assume that because these products are “natural,” they also are safe to use. This assumption may not always be correct, however. For example, chamomile, echinacea, and valerian commonly are used as sleep aids, and like prescription and OTC products that cause sedation, these herbal products may produce enhanced sedative effects in the CNS when combined with alcohol. In addition, liver toxicities caused by various natural products have now been identified (Heathcote and Wanless 1995), and their combination with alcohol may enhance potential adverse effects. To date, limited documentation of such interactions exists because of a lack of scientific studies on this subject (Miller 1998).

## Conclusions

Alcohol’s effects on the metabolism and activities of various medications have been well documented in chronic heavy drinkers. The effects of moderate alcohol consumption, however, have not been studied as thoroughly. Those effects most likely to be clinically significant are the risk of over-sedation resulting from the combination of benzodiazepines and alcohol and the interaction of alcohol with warfarin. Given the variety and complexity of observed interactions between alcohol and numerous medications, it is difficult to recommend an alcohol consumption level that can be considered safe when taking medications. As a rule, people taking either prescription or OTC medications should always read the product warning labels to determine whether possible interactions exist. Similarly, health care providers should be alert to the potential for moderate alcohol use to either enhance medication effects or interfere with the desired therapeutic actions of a medication.

## Figures and Tables

**Table 1:** Alcohol Content of Prescription and Over-the-Counter Medications

Product	Alcohol Content (%)
Betadine (mouthwash, gargle)	8.8
Cepacol (mouthwash, gargle)	14.0
Cheracol Sore Throat	12.5
Chlortrimeton syrup	7.0
Cimetidine Oral Solution	2.8
Cyclosporine Oral Solution	9.5–12.5
Cyproheptadine	5.0
DentSure (denture rinse, peppermint)	14.4
Dr. Tichenor’s Antiseptic	70*
Digoxin Elixir	10.0
Entex	5.0
Furosemide Liquid	11.5
Intensol (diazepam)	19.0
Listerine	26.9
Listerine Cool Mint or Freshburst	21.6
Lomotil Liquid	15.0
Mellaril/Thioridiazine	3.0–4.2
Mentadent Mouthwash	10.0
Oral-B Anti-Plaque Rinse	8.0
Plax-Advanced Formula	8.7
Peri-colace	10.0
Phenobarbital Elixir	14.0
Promethazine/Phenergan	7.0
Ranitidine	7.5
Scope, Baking Soda	9.9
Scope, Cool Peppermint	14.0
Senokot Syrup	7.0
Targon Smokers’ Mouth Wash, Clean Taste	15.6*
Targon Smokers’ Mouth Wash, Original	16.0*
Tavist Oral Solution	5.5
Theophylline Elixir	20.0
Viadent Oral Rinse	10.0

* Specifically denatured alcohol (SDA) 38B content.

SOURCE: Knodel, L.C., ed. *Nonprescription Products: Formulations and Features. 1998–1999.* Washington, DC: American Pharmaceutical Association, 1999.

**Table 2:** Commonly Used Medications That Cause Disulfiram-Like Reactions (i.e., Flushing, Nausea, Vomiting, Sweating) After Alcohol Consumption

Type of Medication	Generic Names	Brand Names
Analgesics (NSAIDs)	Phenacetin	various
	Phenylbutazone	
Antibiotics	Cefamandole	Mandol
	Cefoperazone	Cefobid
	Cefotetan	Cefotan
	Chloramphenicol	various
	Griseofulvin	Fulvicin, Grifulvin,Grisactin
	Isoniazid	Nydrazid, Rifamate, Rifater
	Metronidazole	Flagyl
	Nitrofurantoin	Furadantin, Macrodantin
	Sulfamethoxazole	Bactrim, Septra
	Sulfisoxazole	Pediazole
Cardiovascular medications (nitrates)	Isosorbide dinitrate	Dilatrate, Isordil, Sorbitrate
	Nitroglycerin	Nitro-Bid, Nitrostat
Diabetes medications (sulfonylureas)	Chlorpropamide	Diabinese
	Glyburide	DiaBeta, Glynase, Micronase
	Tolazamide	generic
	Tolbutamide	generic

**Table 3:** Interactions Between Alcohol and Various Classes of Medications

Drug Class (Conditions for which they are used)	Generic Name	Brand Name	Availability	Type of Interaction
Analgesics (pain relief)	Aspirin	various	Rx and OTC	Aspirin increases gastric emptying, leading to faster alcohol absorption in the small intestine; may also inhibit gastric ADH. Alcohol enhances acetaminophen metabolism into a toxic product, potentially causing liver damage.
	Acetaminophen	e.g., Tylenol		
Antibiotics (microbial infections)	Erythromycin	various	Rx	Erythromycin may increase gastric emptying, leading to faster alcohol absorption in the small intestine. Alcohol increases the risk of isoniazid-related liver disease.
	Isoniazid	Nydrazid, Rifamate, Rifater		
Anticonvulsants (seizure disorders)	Phenytoin	Dilantin	Rx	Chronic alcohol consumption induces phenytoin breakdown.
Antihistamines (allergies, colds)	Diphenhydramine	e.g., Benadryl	Rx and OTC	lcohol enhances the effects of these agents on the central nervous system (CNS), such as drowsiness, sedation, and decreased motor skills. The interactions are more pronounced in elderly people. No documented interactions exist with nonsedating antihistamines (i.e., certrizine, hismanal, loratidine).
	Chlorpheniramine Clemastine	various		
	Hydroxyzine	Atarax, Vistaril		
	Promethazine	Phenergan		
	Cyproheptadine	Periactin		
Anticoagulants (prevention of blood clots)	Warfarin	Coumadin	Rx	Acute alcohol intake may increase anticoagulation by decreasing warfarin metabolism; chronic alcohol ingestion decreases anticoagulation by increasing warfarin metabolism.
Antidiabetic agents (blood sugar regulation)	Chlorpropamide	Diabinese	Rx	Alcohol consumption by diabetic patients taking these medications increases the risk of lower-than-normal blood sugar levels (i.e., hypoglycemia). Chlorpropamide, glyburide, and tolbutamide can cause disulfiram-like interactions after alcohol ingestion. Metformin may cause increased levels of lactic acid in the blood after alcohol consumption.
	Glipizide	Glucotrol		
	Glyburide	DiaBeta, Glynase, Micronase		
	Tolbutamide	Orinase		
	Metformin	Glucophage		
Barbiturates (anesthesia, pain relief)	Phenobarbital	various	Rx	Chronic alcohol intake increases barbiturate metabolism by cytochrome P450. Alcohol enhances the sedative and hypnotic effects on the CNS.
Benzodiazepines (sedative agents)	Alprazolam	Xanax	Rx	Alcohol enhances the effects of these agents on the CNS, such as drowsiness, sedation, and decreased motor skills.
	Chlordiazepoxide	Librium		
	Clonazepam	Klonopin		
	Clorazepate	Tranxene		
	Diazepam	Valium		
	Lorazepam	Ativan		
	Midazolam	Versed		
	Oxazepam	Serax		
	Temazepam	Restoril		
	Triazolam	Halcion		
Histamine H_2_ receptor antagonists (ulcers, heart burn)	Cimetidine	Tagamet	Rx and OTC	The agents inhibit ADH in the stomach, thereby reducing alcohol first-pass metabolism (see figure 1), as well as increase gastric emptying. As a result, BALs are higher than expected for a given alcohol dose; this effect increases over time.
	Nizatidine	Axid		
	Ranitidine	Zantac		
Immune modulators (rheumatoid arthritis)	Methotrexate	Rheumatrex	Rx	Immune modulators (i.e., medications that affect immune cell function) are associated with a risk of liver damage, which is increased in combination with alcohol.
Muscle relaxants	Carisoprodol	Soma	Rx	Alcohol consumption enhances impairment of physical abilities (e.g., driving) and increases sedation. Carisoprodol produces an opiate-like high when taken with alcohol; it is metabolized to meprobamate and sometimes abused as a street drug.
	Cyclobenzaprine	Flexeril		
NSAIDs (pain relief and inflammation)	Ibuprofen	e.g., Motrin	Rx and OTC	Alcohol consumption increases the associated risk of gastrointestinal bleeding.
	Flurbiprofen	various		
	Fenoprofen	Nalfon		
	Ketoprofen	Orudis		
	Naproxen	Naprosyn		
	Diclofenac	Voltaren		
Opioids (pain relief)	Codeine	various	Rx	Alcohol enhances the effects of these agents on the CNS, such as drowsiness, sedation, and decreased motor skills.
	Hydromorphone	Dilaudid		
	Fentanyl	generic		
	Morphine	various		
	Meperidine	e.g., Demerol		
	Propoxyphene	Darvon, Wygesic		
Sedatives and hypnotics	Chloral hydrate	Noctec	Rx	Alcohol inhibits the metabolism of these agents and produces a depressant effect on the CNS that includes sleepiness, disorientation, incoherence, and confusion.
	Meprobamate	Equanil, Miltown		
Tricyclic antidepressants (depression)	Amitriptyline	Elavil, Endep	Rx	Alcohol consumption increases the risk of sedation and a sudden drop in blood pressure when a person stands up (i.e., orthostatic hypotension).
	Clomipramine	Anafranil		
	Desipramine	Norpramin		
	Doxepin	Adapin, Sinequan		
	Imipramine	Tofranil		
	Nortriptyline	Aventyl, Pamelor		
	Trimipramine	Surmontil		
Herbal medications (sleep aids)	Chamomile	various	OTC	Alcohol may accentuate the drowsiness that is associated with these herbal preparations.
	Echinacea	preparations		
	Valerian			

ADH = alcohol dehydrogenase; BAL = blood alcohol level; NSAIDs = nonsteroidal anti-inflammatory drugs; OTC = over the counter; Rx = prescription.

## Footnotes

1 A standard drink is defined as one 12-ounce can of beer or bottle of wine cooler, one 5-ounce glass of wine, or 1.5 ounces of distilled spirits and is equivalent to approximately 0.5 ounce, or 12 grams (g), of pure alcohol.

2 Low alcohol doses are defined here as 0.3 g per kilogram body weight, equivalent to approximately two standard drinks for a person weighing 70 kg.

3 The endoplasmic reticulum is an extensive network of membrane-enclosed tubules within the cell that is involved in the production of proteins and fat molecules and in the transport of those molecules within the cell.

4 An Internet Web site (www.accp.com/p450.html) catalogs the classes of cytochrome P450 molecules that can metabolize various medications. This resource can help identify medications metabolized by CYP2E1 that may potentially interact with alcohol.

5 Another class of medications, which prevent gastric acid production through a different mechanism from the H_2_RAs (i.e., omeprazole and lansoprazole), also do not appear to interact with alcohol.

6 Moderate alcohol use by itself, however, does not appear to be associated with an increased risk of ulcers or gastrointestinal bleeding and also is unlikely to cause a certain type of inflammation of the stomach lining (i.e., hemorrhagic gastritis) that has been observed after heavy alcohol use.
